# Pan-cancer multi-omics profiling of MS4A2 unveils its functional landscape in lung adenocarcinoma

**DOI:** 10.1097/JS9.0000000000002903

**Published:** 2025-07-02

**Authors:** Bitian Zhang, William Chi-Shing Cho, Ping-Chung Leung, Chun-Kwok Wong, Dongjie Wang

**Affiliations:** a Institute of Chinese Medicine and State Key Laboratory of Research on Bioactivities and Clinical Applications of Medicinal Plants, The Chinese University of Hong Kong, Hong Kong, China; b Department of Clinical Oncology, Queen Elizabeth Hospital, Hong Kong China; c Department of Chemical Pathology, The Chinese University of Hong Kong, Prince of Wales Hospital, Hong Kong, China; d Li Dak Sum Yip Yio Chin R & D Centre for Chinese Medicine, The Chinese University of Hong Kong, Hong Kong, China; e Shenzhen Research Institute, The Chinese University of Hong Kong, Shenzhen, China

**Keywords:** lung adenocarcinoma, mast cell, MS4A2, pan-cancer, tumor microenvironment

## Abstract

**Background:**

The membrane-spanning 4-domains subfamily A member 2 (MS4A2), a mast cell-specific IgE receptor component, exhibits paradoxical roles in cancer progression. While pan-cancer analyses suggest its context-dependent duality, its prognostic hierarchy across survival metrics – overall survival (OS), disease-specific survival (DSS), progression-free interval (PFI), and disease-free interval (DFI) – remains uncharacterized in lung adenocarcinoma (LUAD).

**Materials and methods:**

We integrated bulk RNA-seq (TCGA/GTEx, 33 cancers) and single-cell transcriptomics (NSCLC cohorts) to map MS4A2’s expression dynamics. Prognostic impacts were quantified through Cox regression and Kaplan–Meier modeling for all four survival endpoints (OS/DSS/PFI/DFI), complemented by immune infiltration and methylation-epigenetic correlation analyses. Drug sensitivity associations were evaluated using GDSC pharmacogenomics.

**Results:**

Pan-cancer stratification revealed LUAD-specific prognostic dominance of MS4A2, with elevated expression reducing mortality risk (OS HR = 0.58, DSS HR = 0.61; all *P* < 0.05) and prolonging disease control (PFI HR = 0.59, DFI HR = 0.59; all *P* < 0.05). Single-cell mapping localized MS4A2 to tumor-associated mast cells, where its bidirectional chemokine regulation – suppressing lymphoid homing (CCL19/CCL21) while amplifying myeloid recruitment (CCL20/CXCL8) – orchestrated leukocyte transendothelial migration. Clinically, MS4A2 exhibited female-biased expression (*P* < 0.01) and stage-dependent attenuation (*P* < 0.01), correlating with enhanced sensitivity to 5-fluorouracil and axitinib (*P* < 0.05) in high-expressing tumors despite their immune-evasive traits.

**Conclusion:**

By converging multi-omics evidence across four prognostic axes (OS/DSS/PFI/DFI), we redefine MS4A2 as a mast cell-driven gatekeeper of LUAD progression. Its dual chemokine polarization establishes an immunosuppressive niche paradoxically susceptible to cytotoxic agents, proposing a precision stratification framework: MS4A2 high tumors for 5-FU/axitinib regimens combined with immunotherapies to counterbalance microenvironmental resistance. This quad-metric prognostic model advances LUAD management by linking mast cell biology to clinically actionable survival endpoints.

## Introduction

Globally, cancer remains a major public health challenge, with the latest 2024 statistics indicating a continued rise in incidence and mortality rates^[1]^. 19.3 million cancer cases were newly diagnosed in 2020 and this population will increase to 29.4 million worldwide in 2040^[2]^. Amidst this escalating burden, molecular drivers exhibiting tissue-specific oncogenic duality – particularly those modulating immune-microenvironment crosstalk-demand urgent investigation. The MS4A2 gene, encoding the beta subunit of the high-affinity IgE receptor (FcεRIβ)^[3-5]^, drives hypersensitivity through IgE-dependent activation^[6,7]^. While its canonical role in asthma^[8]^ and anaphylaxis^[9]^ is well characterized, emerging evidence positions MS4A2 at the crossroads of immune dysregulation^[10]^ and oncogenesis. Paradoxically, this archetypal mediator of type I hypersensitivity demonstrates histotype-specific duality in cancer biology^[11]^ – exhibiting tumor-suppressive effects in adreno cortical carcinoma (ACC) yet accelerating metastatic progression in gastric malignancies^[12]^. Such discordant observations underscore a fundamental question: how does a mast cell-specific protein exert opposing roles across tumor lineages, and could these context-dependent interactions inform surgical risk stratification or therapeutic decision-making?

Despite advances in understanding mast cells as dual regulators of tumor immunity – balancing anti-neoplastic surveillance with pro-metastatic niche formation – the mechanistic basis of MS4A2’s functional plasticity remains unresolved. Critical knowledge gaps persist, including the lack of pan-cancer expression profiles to delineate its stage- and gender-specific regulation, incomplete characterization of its crosstalk with immune checkpoint networks and cancer stemness pathways, and unexplored potential as a biomarker for predicting surgical outcomes such as recurrence or therapeutic resistance. These limitations hinder clinical translation, particularly given preliminary evidence linking MS4A2 to epithelial-mesenchymal transition (EMT) modulation and tertiary lymphoid structure formation – processes directly relevant to intraoperative challenges like tissue friability and postoperative metastasis.

Resolving these questions carries profound implications for surgical oncology. Clinically, mast cell infiltration correlates with intraoperative hemorrhage risk in lung adenocarcinoma resections^[13,14]^, suggesting that MS4A2’s regulatory role in mast cell activation could refine preoperative risk assessment. Therapeutically, its dual associations with chemokine networks and leukocyte trafficking pathways propose actionable targets for adjuvant therapies to mitigate postoperative immunosuppression. By systematically mapping MS4A2’s genomic, epigenetic, and immunologic landscapes across malignancies, this study bridges mast cell biology and oncologic surgery, offering a paradigm to repurpose IgE pathway inhibitors for cancer management while advancing precision strategies for margin assessment and recurrence surveillance. Of note, artificial intelligence (AI) tools were utilized exclusively for linguistic improvement in the drafting of this manuscript, with full adherence to the transparency guidelines outlined in the TITAN framework^[15]^.

## Material and methods

### MS4A2 expression analysis

The mRNA and protein expression profiles of MS4A2 in human normal tissues were assessed. For tumor tissues, the “Gene_DE” module was employed to evaluate MS4A2 expression at both mRNA and protein levels. Additionally, MS4A2 gene expression data were obtained from the GTEx and TCGA databases. Visualization of results was performed using the R platform.

### Survival analysis

Survival analysis was performed based on the previous protocol^[16]^. Generally, the association between MS4A2 expression and patient survival outcomes – including overall survival (OS), disease-specific survival (DSS), disease-free interval (DFI), and progression-free interval (PFI) – was analyzed using the Rstudio platform. Kaplan–Meier curves were generated via the Pan-cancer module by inputting the gene name and selecting the relevant survival metric. Hazard ratios were calculated to further quantify the relationship.

**HIGHLIGHTS**

- MS4A2 demarcates LUAD as the pan-cancer outlier with quad-survival protection (OS/DSS/PFI/DFI HR = 0.58–0.61; *P* ≤ 0.01), the only malignancy achieving cross-endpoint significance.
- Single-cell transcriptomics delineates mast cell-specific MS4A2 expression exhibiting significant covariation (*P* < 0.05) with leukocyte transendothelial migration capacity in LUAD, concomitant with chemokine axis rewiring: inverse CCL19 (r = 0.32)/CCL21 (r = 0.12) vs. direct CCL20 (r = −0.205)/CXCL8 (r = −0.141) associations.
- LUAD-specific sexual dimorphism emerges as MS4A2 shows female-enriched expression (*P* < 0.01) with progressive attenuation across stages (*P* < 0.01), indicating orthogonal regulatory axes in tumor evolution.
- MS4A2 predicts therapeutic vulnerability in LUAD through endothelial barrier regulation and drug sensitivity significance of 5-Fluorouracil and axitinib (*P* < 0.05).

### Correlation analysis of MS4A2 expression with clinical parameters

The Timer database was utilized to explore correlations between MS4A2 gene expression and clinical parameters, including cancer stage, TNM stage, gender, and age. Subtype-specific MS4A2 expression patterns were further analyzed across significant cancer types.

### Pan-cancer mutation landscape analysis

An oncoplot was generated to depict the mutational status of MS4A2 across various tumors. Given the significant association of MS4A2 expression with clinical outcomes in LUAD, stomach adenocarcinoma (STAD), head and neck squamous cell carcinoma (HNSC), and ACC, these cancer types were prioritized for mutation landscape analysis. The Geneset Cancer Analysis (GSCA) database was employed to examine single nucleotide variations (SNVs), copy number variations (CNVs), and methylation patterns of MS4A2, alongside their relationship with mRNA expression levels. The “Mutation” module was used to assess correlations between mRNA expression and CNV or methylation status.

### Correlation analysis of MS4A2 expression with cancer stemness

Given the critical role of cancer stem cells in tumor recurrence, metastasis, and therapeutic resistance, the association between MS4A2 expression and tumor stemness was evaluated. Additionally, the correlation between MS4A2 expression and biomarkers of epithelial-mesenchymal transition (EMT) – a key process in tumorigenesis and metastasis – was analyzed. Heatmaps depicting these relationships were generated using RStudio software (version 4.4.0).

### Immune infiltration analysis

Stromal, immune, and ESTIMATE scores were computed using the tumor infiltration function on the Sangerbox 3.0 platform (http://sangerbox.com/home.html). A correlation heatmap was generated to visualize these relationships using RStudio software (version 4.4.0). Pan-cancer immune infiltration analysis was conducted to assess MS4A2 expression across immune cell types, including B cells, T cells, neutrophils, macrophages, and dendritic cells. Tumor mutational burden (TMB) and microsatellite instability (MSI) scores were also calculated in relation to MS4A2 expression.

### Correlation analysis of MS4A2 with chemokine expression

A comprehensive list of chemokines was compiled, and scatter plots were generated to evaluate the relationship between MS4A2 and chemokine expression using the Xiantaozi database (www.xiantaozi.com).

### Correlation analysis of MS4A2 expression with drug sensitivity

The association between MS4A2 expression and chemotherapeutic drug sensitivity was evaluated using the Genomics of Drug Sensitivity in Cancer (GDSC) database via the Gene Set Cancer Analysis (GSCA) platform to predict half-maximal inhibitory concentration (IC_50_) values. Correlation analyses were visualized using scatter plots generated in RStudio. For cancer types demonstrating statistically significant associations, IC_50_ values were further compared between tumors with high versus low MS4A2 expression levels.

### Pearson correlation analysis

Pearson correlation analysis was performed in lung cancer using R (Version 4.4.0).

### Enrichment analysis of MS4A2-associated genes

In lung adenocarcinoma (LUAD), the regulatory mechanisms of MS4A2-associated genes were explored through Gene Ontology (GO) biological processes and KEGG pathway analyses using R (Version 4.4.0).

### Single-cell RNA sequencing analysis of MS4A2

Single-cell RNA sequencing data were obtained from the cellxgene database (https://cellxgene.cziscience.com/collections) to investigate MS4A2 expression across diverse cell types in various cancers. Correlation graphs depicting cell type-specific MS4A2 expression were generated using the TISCH online database (http://tisch.comp-genomics.org/)^[17]^.

## Statistical analysis

All statistical analyses were performed using appropriate biostatistical methods, including Pearson’s correlation test, ANOVA, and t-tests. A *P*-value threshold of <0.05 was used to determine statistical significance. The statistical analyses were performed in R (Version 4.4.0).

The data sources and their applications are summarized in Supplemental Digital Content, Table 1 (available at: http://links.lww.com/JS9/E576).

## Results

### Pan-cancer expression dynamics of MS4A2 prioritizing prognostic significance in lung adenocarcinoma

**Figure 1.**
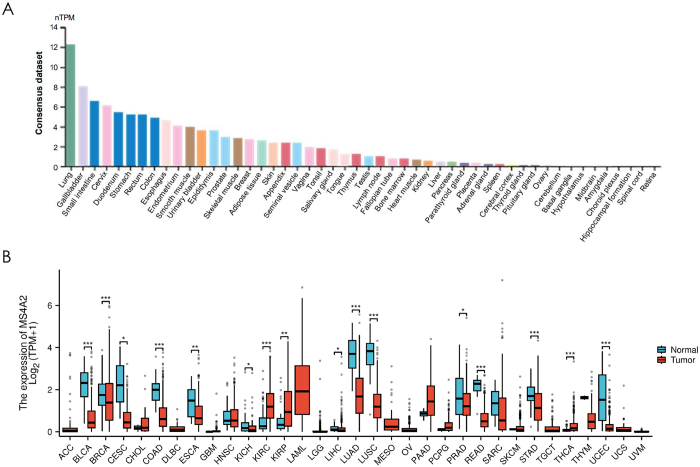
Visualizing MS4A2 expression in normal tissues and comparing it in pan-cancer settings. (A) Normal tissue landscape of MS4A2 Expression: The y-axis represents normalized transcript per million (nTPM) values, a metric for gene expression levels. The x-axis lists different normal tissues. The bar chart effectively demonstrates the wide-ranging expression levels of MS4A2 across tissues, highlighted by the prominent expression in the lung. (B) Pan-cancer assessment of MS4A2 expression: a comparison between normal and tumor tissues. The y-axis shows the log-transformed expression values of MS4A2 (log(TPM + 1)), while the x-axis enumerates various cancer types, abbreviated as follows: ACC, BLCA (bladder urothelial carcinoma), BRCA (breast invasive carcinoma), CESC (cervical squamous cell carcinoma and endocervical adenocarcinoma), CHOL (cholangiocarcinoma), COAD, DLBC (lymphoid neoplasm diffuse large B-cell lymphoma), ESCA (esophageal carcinoma), GBM (glioblastoma multiforme), HNSC, KIRC, KIRP (kidney renal papillary cell carcinoma), LAML (acute myeloid leukemia), LGG (brain lower-grade glioma), LIHC (liver hepatocellular carcinoma), LUAD (lung adenocarcinoma), LUSC (lung squamous cell carcinoma), MESO (mesothelioma), OV (ovarian serous cystadenocarcinoma), PAAD, PCPG (pheochromocytoma and paraganglioma), PRAD (prostate adenocarcinoma), READ (rectum adenocarcinoma), SARC (sarcoma), SKCM (skin cutaneous melanoma), STAD, TGCT (testicular germ cell tumors), THCA (thyroid carcinoma), THYM (thymoma), UCEC (uterine corpus endometrial carcinoma), UCS (uterine carcinosarcoma), and UVM (uveal melanoma). The box-and-whisker plots illustrate the distribution of gene expression in normal (teal) and tumor (red) tissues for each cancer type. Asterisks indicate the statistical significance of the expression difference between normal and tumor tissues (*P < 0.05, **P < 0.01, ***P < 0.001).

MS4A2 expression demonstrates marked tissue-specific heterogeneity. Analysis of transcriptomic data revealed the highest MS4A2 expression in the lung and gallbladder, with normalized transcript abundance (nTPM) exceeding 10, while moderate levels were detected in bladder, colon, esophagus, and stomach tissues (Fig. 1A). In contrast, many other tissues, including kidney, liver, pancreas, and brain regions, showed minimal or undetectable MS4A2 expression.

In paired comparisons of normal versus tumor tissues (Fig. 1B), MS4A2 expression was significantly downregulated in lung adenocarcinoma (LUAD), bladder urothelial carcinoma, and several gastrointestinal cancers. Conversely, elevated MS4A2 levels were observed in kidney renal clear cell carcinomas (KIRC). These findings highlight the context-dependent regulation of MS4A2 across different tumor types, suggesting a potential role in tissue-specific tumor biology and immune modulation. We also investigated MS4A2 gene expression in normal tissue using another two databases (HPA and GTEx databases) and both of these showed highest MS4A2 gene expression in lung tissue (Supplementary Digital Content, Figure 1, available at: http://links.lww.com/JS9/E575).

**Figure 2.**
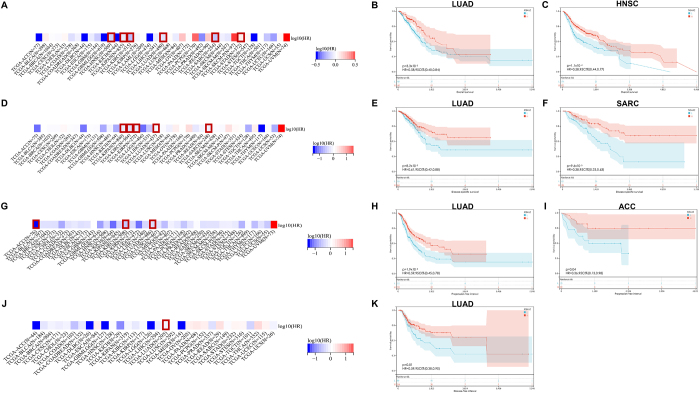
Pan-cancer prognostic analysis of MS4A2 gene. (A) Pan-cancer correlation analysis of the MS4A2 gene with overall survival (OS). Red-boxed cancers indicate significant associations (*P* < 0.05) between MS4A2 and OS. (B, C) Kaplan–Meier (KM) curves of OS for representative cancers. B shows the KM curve for lung adenocarcinoma (LUAD), and C shows the curve for HNSC. (D) Pan-cancer correlation analysis of the MS4A2 gene with disease-specific survival (DSS). Red-boxed cancers denote significant correlations (*P* < 0.05) between MS4A2 and DSS. (E, F) KM curves of DSS for representative cancers. Panel E presents the results for LUAD, and Panel F shows the curve for sarcoma (SARC). (G) Pan-cancer correlation analysis of the MS4A2 gene with progression-free interval (PFI). Red-boxed cancers imply significant associations (*P* < 0.05) between MS4A2 and PFI. (H, I) KM curves of PFI for representative cancers. H shows the curve for LUAD, and I presents the results for ACC. (J) Pan-cancer correlation analysis of the MS4A2 gene with disease-free interval (DFI). Red-boxed cancers represent significant correlations (*P* < 0.05) between MS4A2 and DFI. (K) KM curve of DFI for LUAD. The KM curves display the survival probability differences between high and low MS4A2 expression groups, while the pan-cancer correlation plots identify the specific cancer types in which MS4A2 is significantly associated with distinct survival endpoints.

Survival analyses further substantiated this functional dichotomy. Multivariate Cox regression across 33 cancer types identified LUAD as a consistent beneficiary of high MS4A2 expression, showing improved overall survival (OS, HR = 0.77, *P* < 0.01) (Fig. 2A), disease-specific survival (DSS, HR = 0.76, *P* < 0.05) (Fig. 2D), progression-free interval (PFI, HR = 0.78, *P* < 0.01) (Fig. 2G), and disease-free interval (DFI, HR = 0.75, *P* < 0.05) (Fig. 2J). This protective association was validated through Kaplan–Meier stratification, where LUAD patients with elevated MS4A2 exhibited greater 5-year survival probability (*P* < 0.01) (Fig. 2B). While similar benefits extended to HNSC (OS, HR = 0.58) (Fig. 2C) and sarcomas (SARC, DSS, HR = 0.38) (Fig. 2F) and ACC (PFI, HR = 0.36) (Fig. 2I), highlighting its context-dependent prognostic duality. Notably, temporal endpoint analyses solidified LUAD-specific consistency, with high MS4A2 expression predicting prolonged disease-free interval (DFI, HR = 0.59) (Fig. 2K). Apart from these LUAD specific survival curve, MS4A2 gene expression is also correlated with other cancer types such as kidney cancers, SARC, STAD. In these cancer types, MS4A2 also showed bidirectional regulatory properties (Supplementary Digital Content, Figure 2, available at: http://links.lww.com/JS9/E575).

### Pan-cancer transcriptomic convergence nominates MS4A2 as a stage-sex stratified biomarker in LUAD

**Figure 3.**
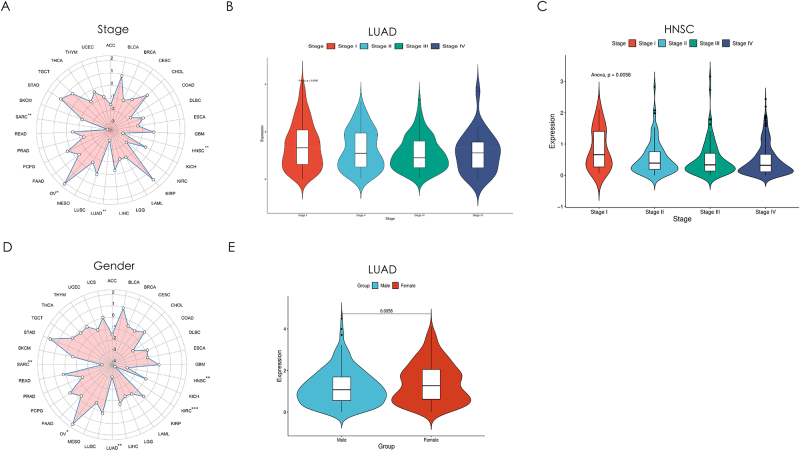
Clinical-demographic associations across cancer types. (A) A radar plot showing the correlation between MS4A2 expression and cancer stage across multiple cancer types. Cancer types with a significant correlation are marked with asterisks (*) on the right-hand side of the cancer names. Cancers marked with ** indicate *P* < 0.01. (B) Correlation analysis of MS4A2 expression and cancer stage in Lung Adenocarcinoma (LUAD). A significant inverse relationship was detected (*P* = 0.0097). Although MS4A2 expression generally decreased as the stage advanced, there was a slight increase in stage 4. (C) Association of MS4A2 expression with cancer stage in HNSC. A significant negative correlation was found (*P* = 0.0056), with MS4A2 expression decreasing as the stage increased. (D) A radar plot showing the correlation between MS4A2 expression and gender across multiple cancer types. Cancer types with a significant correlation are marked with asterisks (*) on the right-hand side of the cancer names. Cancers marked with * indicate *P* < 0.05; cancers marked with ** indicate *P* < 0.01; cancers marked with *** indicate *P* < 0.001. (E) Comparison between MS4A2 gene expression versus patient genders.

Systematic evaluation of MS4A2’s clinical relevance revealed cancer-type-specific progression patterns, with a particular focus on LUAD. Pan-cancer analysis showed a significant inverse correlation between MS4A2 expression and tumor stage in LUAD and HNSC, contrasting with a positive association in ovarian cancer (Fig. 3A). In LUAD, stage-stratified analysis revealed a consistent decline in MS4A2 expression across disease progression (Stage I to Stage III, *P* < 0.01), suggesting potential compensatory mechanisms in advanced stages (Fig. 3B). HNSC exhibited a similar trend, while ovarian cancer showed no stage-dependent regulation, highlighting lineage-specific regulatory divergence (Fig. 3C).

MS4A2 exhibited pan-cancer age-associated suppression, with significant negative correlations in LUAD, HNSC, KIRC, and SARC cohorts (global cohort analysis, *P* < 0.01; Supplementary Digital Content, Figure 3A, available at: http://links.lww.com/JS9/E575). However, age-stratified subgroup analyses revealed organ-specific divergences: LUAD lost this age-dependent regulation when partitioned into young/elderly subgroups (*P* > 0.05), whereas KIRC and STAD maintained robust MS4A2 suppression in elderly patients (*P* < 0.01; Supplementary Digital Content, Figure 3C, F, G, and H, available at: http://links.lww.com/JS9/E575).

Gender-specific analysis revealed sexual dimorphism in MS4A2 regulation (Fig. 3D), with LUAD exhibiting significant female-biased expression (*P* < 0.01) (Fig. 3E). Other cancers, including HNSC, KIRC, and SARC, showed gender-neutral profiles (Supplementary Digital Content, Figure 4A–C, available at: http://links.lww.com/JS9/E575). This LUAD-specific sexual dimorphism, coupled with its age-independent regulation, points to unique pulmonary-specific epigenetic or transcriptional control mechanisms.

### Tumor microenvironment interactions of MS4A2 from pan-cancer landscapes to LUAD-specific immune modulation

**Figure 4.**
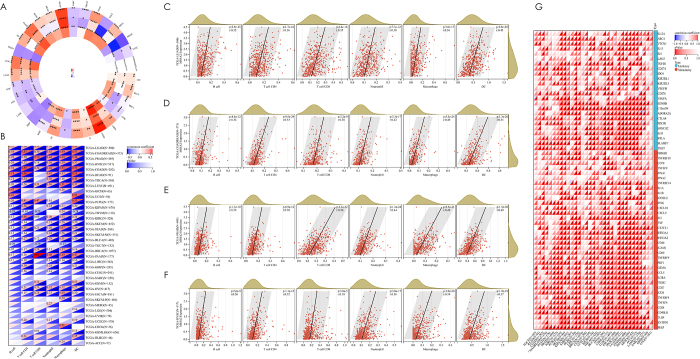
Pan-cancer analysis of the association between MS4A2 expression, tumor microenvironment scores, and immune cell infiltration. (A) Circular heatmap showing the correlation between MS4A2 expression and stromal score, immune score, and ESTIMATE score across pan-cancer types. The outer, middle, and inner circles represent stromal score, immune score, and ESTIMATE score, respectively. Red squares indicate positive correlations, while blue squares denote negative correlations. Statistical significance is denoted by asterisks (*P < 0.05; **P < 0.01; ***P < 0.001; ****P < 0.0001). (B) Heatmap illustrating the correlation between MS4A2 expression and immune cell infiltration, including B cells, CD4^+^ T cells, CD8^+^ T cells, neutrophils, macrophages, and dendritic cells, across 33 cancers. Red regions indicate positive correlations, whereas blue represents negative correlations. Statistical significance is denoted by asterisks (*P < 0.05; **P < 0.01; ***P < 0.001; ****P < 0.0001). Cells without markings indicate nonsignificant correlations. (C-F) Scatterplots depicting the correlation between MS4A2 expression and immune cell infiltration in selected representative cancer types: (C) LUAD (n = 500), (D) COADREAD (n = 373), (E) PRAD (n = 495), and (F) HNSC (n = 517). MS4A2 expression shows significant positive correlations with B cells, CD4^+^ T cells, CD8^+^ T cells, neutrophils, macrophages, and dendritic cells in all four cancers. (G) Heatmap depicting the correlation between MS4A2 and immune checkpoint genes (both inhibitory and stimulatory). Positive (red) and negative (blue) correlations are indicated, with statistical significance marked by asterisks (*). The rightmost columns summarize average correlations for stimulatory (red) and inhibitory (blue) checkpoint molecules.

Pan-cancer interrogation of MS4A2’s tumor-ecosystem interactions revealed a dual regulatory paradigm. Compartment-specific microenvironment modulation was described in dimensions of StromalScore, ImmuneScore and ESTIMATEScore (Fig. 4A). This dichotomy resolved through lineage-specific analysis: LUAD, PRAD, and HNSC demonstrated MS4A2-associated pan-immune cell infiltration (CD4^+^ T cells: r = 0.36, CD8^+^ T cells: r = 0.35, B cells: r = 0.32, macrophages: r = 0.36 in LUAD; CD4^+^ T cells: r = 0.30, CD8^+^ T cells: r = 0.50, B cells: r = 0.28, macrophages: r = 0.49 in PRAD; CD4^+^ T cells: r = 0.32, CD8^+^ T cells: r = 0.18, B cells: r = 0.26, macrophages: r = 0.39 in HNSC), validated across seven independent cohorts (Fig. 4B-F; Supplementary Digital Content, Figure 5A–G, available at: http://links.lww.com/JS9/E575). Thyroid carcinoma (THCA) emerged as a notable exception, showing MS4A2-CD8^+^ T cell decoupling (r = −0.04, *P* = 0.35), indicative of tissue-contextual immune regulation (Supplementary Digital Content, Figure 5H, available at: http://links.lww.com/JS9/E575).

Mechanistically, MS4A2 orchestrates a complex interplay between stimulatory and inhibitory immune checkpoint networks, as revealed by pan-cancer heatmap profiling (Supplementary Digital Content, Figure 6A, available at: http://links.lww.com/JS9/E575). This bidirectional immunomodulatory role is particularly evident in LUAD, where MS4A2 expression strongly correlates with key immune checkpoints, including PD-1 (r = 0.1344), CTLA-4 (r = 0.2204), and LAG-3 (r = 0.0328), while inversely associating with CCL20 (r = −0.0716) and CXCL8 (r = −0.1285). These associations underscore MS4A2’s pivotal role as a master regulator of immune equilibrium, intricately balancing pro- and anti-tumor immune responses in a context-dependent manner. In lung adenocarcinoma (LUAD), the positive correlations with stimulatory checkpoints such as PD-1 (r = 0.1343), CTLA-4 (r = 0.2204), and LAG-3 (r = 0.0328) suggest MS4A2 enhances immune activation, while its inverse correlations with pro-inflammatory chemokines CCL20 (r = −0.1844) and CXCL8 (r = −0.1285) indicate a concurrent dampening of inflammatory pathways, highlighting its nuanced capacity to modulate both immune activation and suppression within the tumor microenvironment. MS4A2 expression shows context-dependent correlations with genomic instability, with no significant MSI or TMB associations in LUAD, but negative MSI correlation in HNSC and positive in GBM and ovarian cancer, alongside negative TMB correlation in STAD and positive in pheochromocytoma/paraganglioma (Supplementary Digital Content, Figure 6B and C, available at: http://links.lww.com/JS9/E575). These findings highlight MS4A2’s multifaceted role in modulating immune and genomic landscapes across diverse cancer types.

### Convergent epigenetic and genetic mechanisms of MS4A2 dysregulation prioritizing LUAD pathobiology

**Figure 5.**
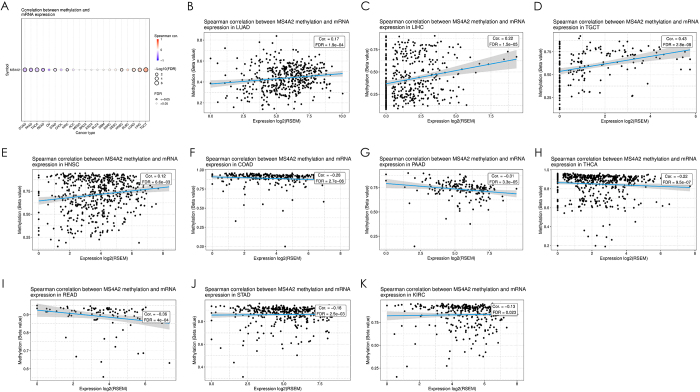
Pan-cancer analysis of the association between MS4A2 expression and DNA methylation. (A) Heatmap summarizing the correlation coefficients between MS4A2 mRNA expression and DNA methylation levels for all cancer types. Blue circles represent negative correlations, while red circles indicate positive correlations. Cancer types with significant correlations (*P* < 0.05) include COAD, PAAD, THCA, READ, STAD, KIRC, HNSC, LUAD, LIHC, and TGCT. (B-K) Regression plots of DNA methylation levels against MS4A2 mRNA expression for the cancer types with significant correlations identified in (A), including (B) LUAD, (C) LIHC, (D) TGCT, (E) HNSC, (F) COAD, (G) PAAD, (H) THCA, (I) READ, (J) STAD, and (K) KIRC. Blue regression lines with shaded confidence intervals quantify the direction and magnitude of correlations. Negative correlations are observed in COAD, PAAD, THCA, READ, STAD, and KIRC, while positive correlations are identified in HNSC, LUAD, LIHC, and TGCT. Statistical significance was determined using Spearman correlation tests.

Pan-cancer epigenetic profiling revealed bidirectional DNA methylation control of MS4A2 expression, governed by tumor lineage (Fig. 5A). Significant CpG hypermethylation-mediated suppression (colon adenocarcinoma (COAD), r = −0.28, FDR<0.0001; STAD: r = −0.16, FDR<0.01) dominated gastrointestinal malignancies and endocrine tumors (pancreatic adenocarcinoma: r = −0.31, FDR<0.0001; thyroid cancer: r = −0.22, FDR<0.0001; KIRC: r = −0.13, FDR<0.05), contrasting with hypomethylation-driven overexpression in aerodigestive cancers (HNSC, r = 0.12, FDR<0.01; LUAD: r = 0.17, FDR<0.001) and hepatic/germ cell tumors (LIHC: r = 0.22, FDR<0.0001; testicular germ cell tumors: r = 0.43, FDR<0.0001). Multi-cohort validation confirmed this tissue-specific regulatory dichotomy: LUAD exhibited progressive methylation correlating with MS4A2 upregulation (r = 0.17, FDR<0.001), whereas STAD showed methylation loss (r = −0.16, FDR<0.01) (Fig. 5B-K).

Genetic interrogation uncovered three tiers of MS4A2 genomic disruption. At the mutation level, skin cancer exhibited the highest SNV burden (4.27%), followed by rectal adenocarcinoma mutation cluster (2.68%). In LUAD, SNV burden accounted for 1.23% co-occurring with TP53 (*P* < 0.01) and KEAP1 (*P* < 0.01) alterations (Supplementary Digital Content, Figure 7A and C, available at: http://links.lww.com/JS9/E575). Copy-number analysis revealed STAD-specific MS4A2 amplification driving transcriptional overexpression (r = 0.12, FDR<0.05), while LUAD maintained CNV-independent regulation (r = −0.03, FDR>0.05) despite high mutation frequency (Supplementary Digital Content, Figure 7B, available at: http://links.lww.com/JS9/E575).

### MS4A2 modulates diverse drug responses in pan-cancer analysis and LUAD-specific therapeutic potential

**Figure 6.**
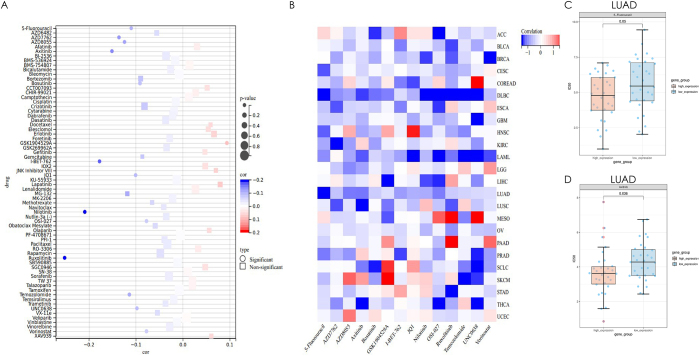
Pan-cancer drug sensitivity analysis of MS4A2 expression. (A) Aggregate analysis of the correlation between MS4A2 expression and drug sensitivity across multiple anticancer agents in pan-cancer datasets. Drug sensitivity outcomes are represented by red (increased resistance with high MS4A2 expression) and blue (increased sensitivity with high MS4A2 expression). Circle markers denote statistically significant correlations, whereas square markers denote non-significant associations. (B) Heatmap illustrating the relationship between MS4A2 expression and the sensitivity to 14 specified drugs in individual cancer types. Red and blue cells reflect resistance and sensitivity, respectively. (C, D) Boxplots comparing log₁₀ IC₅₀ (μM) values for 5-Fluorouracil (C) and Axitinib (D) in LUAD patients stratified by high (red) and low (blue) MS4A2 expression. Statistical significance was determined using a t-test, with *P* = 0.05 for 5-Fluorouracil and *P* = 0.036 for Axitinib.

We investigated the role of MS4A2 gene expression in modulating drug sensitivity across diverse cancer types through a comprehensive pan-cancer analysis. Our findings demonstrate that elevated MS4A2 expression drives heterogeneous drug responses, conferring both heightened sensitivity and resistance depending on the therapeutic agent (Fig. 6A). Notably, high MS4A2 expression was significantly associated with increased sensitivity to 13 drugs, including 5-Fluorouracil, Axitinib, AZD7762, AZD8055, Bosutinib, I-BET-762, OSI-027, UNC0638, JQ1, Nilotinib, Ruxolitinib, Temozolomide, and Vorinostat. In contrast, elevated MS4A2 expression was linked to marked resistance to GSK1904529A.

Further analysis of individual cancer types revealed that MS4A2 expression exerts distinct effects on drug response within specific tumor lineages (Fig. 6B). Among these, lung adenocarcinoma (LUAD) exhibited particularly significant drug sensitivity. Box plot analyses of 5-Fluorouracil and Axitinib IC_50_ values relative to MS4A2 expression levels confirmed that high MS4A2 expression significantly enhances LUAD cell responsiveness to these agents (Fig. 6C and D). Specifically, 5-Fluorouracil displayed a notably lower IC_50_ in the high-MS4A2 expression group compared to the low-expression group (*P* = 0.05). A more robust association was observed for Axitinib, with a highly significant *P* value (*P* = 0.036), underscoring a strong link between MS4A2 expression and enhanced LUAD sensitivity to this drug. Collectively, these findings highlight the pivotal, dual role of MS4A2 in shaping drug responses in a cancer- and agent-specific manner, with pronounced therapeutic implications for LUAD.

### MS4A2 downregulation enhances leukocyte transendothelial migration in lung adenocarcinoma from bulk RNA-seq

**Figure 7.**
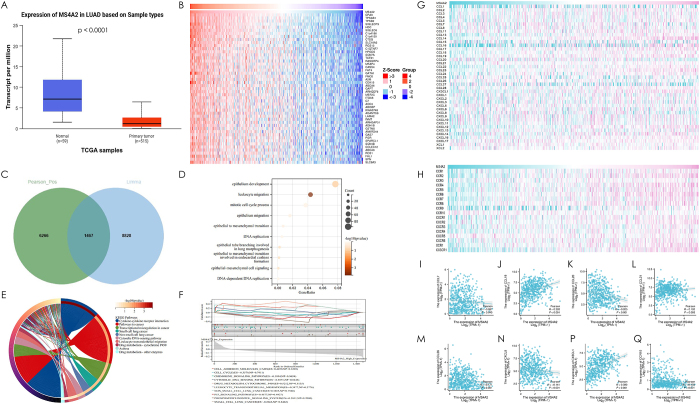
Comprehensive analysis of MS4A2 expression and its correlation with immune-related genes and pathways in LUAD (A) Differential expression of MS4A2 between LUAD tumor tissues (red, n = 515) and normal tissues (blue, n = 59) based on TCGA data (*P* < 0.0001). (B) Heatmap showing representative genes significantly correlated with MS4A2 expression (Pearson correlation analysis). Rows represent genes, and columns represent samples. (C) Venn diagram indicating the intersection of MS4A2-correlated genes from Pearson analysis and DEGs identified via limma analysis, with 1467 shared genes. (D) GO enrichment analysis of the intersecting genes, highlighting “leukocyte migration” as the most enriched biological process. The size and color of the bubble represent the gene count and significance level, respectively. (E) KEGG pathway analysis showing “leukocyte transendothelial migration” as a highly enriched pathway among MS4A2-associated genes. (F) GSEA analysis further corroborating the enrichment of the “leukocyte transendothelial migration” pathway. The normalized enrichment score (NES) reflects the association with this key immune process. (G) Heatmap showing the correlation between MS4A2 expression and chemokine ligands belonging to CCL, CXCL, CX3CL, and XCL families in LUAD. (H) Heatmap showing the correlation between MS4A2 expression and chemokine receptors from the CCR, CXCR, CX3CR, and XCR families in LUAD. (I–Q) Scatter plots illustrating the representative correlations between MS4A2 expression and significantly associated chemokines or receptors: (I) CCL7 (negative correlation, *P* = 0.045), (J) CCL19 (positive correlation, *P* < 0.001), (K) CCL20 (negative correlation, *P* < 0.001), (L) CCL21 (positive correlation, *P* = 0.005), (M) CCL26 (negative correlation, *P* < 0.001), (N) CXCL8 (negative correlation, *P* = 0.001), (P) CX3CL1 (positive correlation, *P* < 0.001), and (Q) CCR10 (negative correlation, *P* < 0.001). Correlations were determined using Pearson’s coefficients.

Integrated analysis of bulk RNA-seq data from lung adenocarcinoma (LUAD) tumors and paired normal tissues revealed that MS4A2 downregulation drives enhanced leukocyte transendothelial migration in LUAD pathogenesis. Comparative transcriptomic profiling between tumor and normal tissues identified significant MS4A2 suppression in LUAD (*P* < 0.0001, Fig. 7A), with its low-expression phenotype stratifying tumors into distinct molecular subgroups. Co-expression network analysis of 1,467 MS4A2-correlated genes revealed robust enrichment in immune cell trafficking pathways (Fig. 7B and C), particularly leukocyte transendothelial migration (Fig. 7D-F). Functional interrogation of MS4A2-associated differentially expressed genes demonstrated its downregulation amplifies lymphocyte migration capacity through conserved pathway activation (leukocyte migration) across clinical subgroups, including stage III–IV vs. I–II and male vs. female cohorts (Supplementary Digital Content, Figure 8, available at: http://links.lww.com/JS9/E575). These findings establish MS4A2 as a master regulator of immune infiltration in LUAD, where its loss triggers endothelial barrier dysregulation to potentiate leukocyte trafficking.

Notably, bulk RNA-seq analysis of MS4A2-stratified LUAD tumors unveiled paradoxical relationships between chemokine networks and immune cell dynamics. MS4A2-low tumors exhibited elevated pro-tumorigenic chemokines CCL20 (r = −0.205, *P* < 0.001), CCL26 (r = −0.171, *P* < 0.001), and CXCL8 (r = −0.141, *P* = 0.001) (Fig. 7K,M,N), contrasted by diminished lymphoid homing chemokines CCL19 (r = 0.320, *P* < 0.001) and CCL21 (r = 0.120, *P* = 0.005) (Fig. 7J and L). This counterintuitive chemokine profile coexisted with enhanced leukocyte transendothelial migration in MS4A2-low tumors (Fig. 6), challenging conventional chemokine-immune axis paradigms.

Mechanistically, two non-mutually exclusive hypotheses emerge. First, ectopic CCR10 suppression in MS4A2-low tumors (r = −0.178, *P* < 0.001, Fig. 7Q) may override CCL20/CXCL8 chemoattraction by disrupting peripheral lymphocyte homing, redirecting immune cells toward tumor parenchyma. Second, residual CCL19/CCL21 signaling in MS4A2-low tumors could sustain lymphocyte recruitment through preferential engagement of high-affinity chemokine receptor isoforms – a compensatory mechanism documented in breast cancer models. These findings suggest MS4A2 downregulation reprograms immune infiltration via spatial redistribution of lymphocyte trafficking rather than linear chemokine concentration changes. While requiring experimental validation through CCR10 knockout models and chemokine receptor isoform mapping, our bulk RNA-seq data position MS4A2 as a critical orchestrator of immune signal prioritization in LUAD.

### Single-cell transcriptomics decoding mast cell dynamics in LUAD

**Figure 8.**
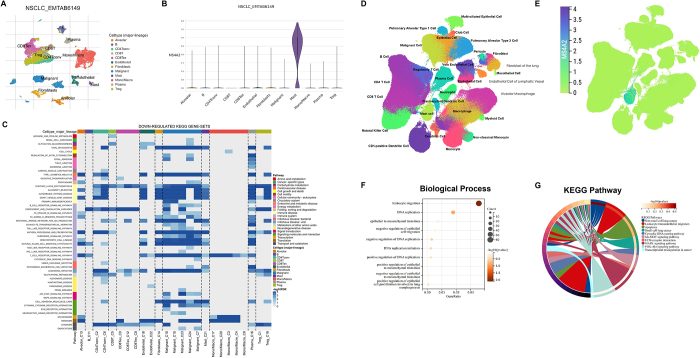
Single-cell transcriptomic analysis reveals mast cell-specific expression of MS4A2 and associated functional pathways. (A) UMAP visualization of major cell types identified in the NSCLC single-cell dataset (EMTAB6149). Cells are color-coded by cluster annotation. (B) Violin plot of MS4A2 expression across different cell types, showing specific enrichment in mast cells. (C) Heatmap of enriched gene sets within mast cells, highlighting significant downregulation of immune-related pathways, including lymphocyte transendothelial migration. (D) UMAP plot of the Salcher_2022_Cancer_Cell dataset, displaying diverse immune and stromal cell populations. (E) Distribution of MS4A2 expression in the UMAP space of Salcher_2022_Cancer_Cell, indicating concentrated expression in mast cells. (F) GO enrichment analysis for mast cells, highlighting pathways such as lymphocyte migration. (G) KEGG pathway analysis of mast cells identifies significant enrichment of lymphocyte transendothelial migration. Together, these panels establish a consistent mast cell-specific expression profile of MS4A2 and its association with immune trafficking modulation across multiple tumor datasets.

To delineate the tumor microenvironment-specific expression patterns of MS4A2, we performed integrated single-cell transcriptomic profiling of non-small cell lung cancer specimens. Unsupervised clustering resolved eight major cellular compartments, including alveolar epithelial cells, T/B lymphocytes, macrophages, endothelial cells, mast cells, fibroblasts, and malignant cells (Fig. 8A). Mast cells exhibited unique MS4A2 expression dominance, with minimal detection in other cell types (Fig. 8B). Functional interrogation revealed significant enrichment of lymphocyte transendothelial migration pathways in mast cells, suggesting their gatekeeper role in immune surveillance (Fig. 8C).

Independent validation through multi-platform single-cell analysis confirmed mast cell-specific MS4A2 expression conservation (Fig. 8D-E). Transcriptomic signatures of these mast cells consistently showed enrichment for lymphocyte migration regulation (Fig. 8F-G). This conserved expression architecture positions mast cells as critical modulators of lymphocyte infiltration dynamics through MS4A2-mediated endothelial barrier regulation.

## Discussion

The interplay between allergic responses and cancer development remains a complex and debated topic in oncology. Our preclinical findings provide the first experimental evidence that allergic inflammation can promote lung carcinogenesis in murine models^[18]^. While MS4A2 (the beta subunit of the high-affinity IgE receptor, FcεRIβ) serves as a central regulator of allergic signaling pathways, its functional role in tumor biology remains incompletely characterized. Notably, MS4A2’s functional impact appears highly context-dependent: In colorectal cancer, tumor microenvironment-derived fibroblasts and endothelial cells regulate MS4A2 expression to suppress tumor progression^[19]^. In LUAD, MS4A2 downregulation correlates with reduced overall survival^[20]^, and its expression serves as an independent prognostic biomarker for early-stage lung cancer^[21]^. However, contrasting roles emerge in gastric cancer: MS4A2 overexpression associates with poorer prognosis despite low expression correlating with improved survival outcomes^[12]^, while aberrant MS4A2 expression predicts lymph node metastasis^[22]^. These paradoxical findings suggest tumor type-specific regulatory mechanisms. Of particular translational significance, MS4A2-encoded FcεRIβ is a critical component in IgE-mediated immune activation. Preclinical studies demonstrate that FcεRI-engineered T cells exert potent anti-tumor effects through IgE-dependent mechanisms^[23]^, highlighting MS4A2 as a potential target for cancer immunotherapy. These findings collectively underscore the necessity for comprehensive investigations into MS4A2’s tumor-specific functions to harness its therapeutic potential.

Employing pan-cancer analysis to address this gap and elucidate the functional role of MS4A2 across malignancies, our integrated bulk and single-cell RNA-Seq analyses ultimately revealed its critical role specifically in LUAD. We establish MS4A2 as a key regulator of mast cell-mediated leukocyte trans-endothelial migration (TEM) in this context, with profound prognostic and therapeutic implications. Bulk RNA-Seq revealed significant MS4A2 downregulation in LUAD tumors compared to normal lung tissue, stratifying tumors into distinct molecular subgroups based on low vs. high MS4A2 expression. Low MS4A2 expression strongly correlated with poor survival outcomes, including reduced OS, DSS, PFI, and DFI. Co-expression network analysis identified robust enrichment for immune cell trafficking pathways, particularly leukocyte TEM, which was markedly upregulated in MS4A2-low tumors^[24]^. Differential expression analysis across clinical subgroups (stage III–IV vs. I–II, male vs. female) consistently showed enrichment of lymphocyte migration (GO) and transendothelial migration (KEGG) pathways, with female-biased MS4A2 expression and stage-dependent hypomethylation further supporting its prognostic relevance. Single-cell RNA-Seq revealed that MS4A2 is predominantly expressed in tumor-associated mast cells, underscoring their pivotal role in orchestrating immune cell infiltration in LUAD^[20]^.

Mechanistically, MS4A2 modulates mast cell-mediated TEM through a dual chemokine network^[25]^. In MS4A2 high-expression tumors, mast cells promote lymphoid homing via elevated expression of chemokines CCL19^[26]^, CCL21^[27]^, and CX3CL1^[28]^, fostering anti-tumor lymphocyte infiltration. Conversely, MS4A2-low tumors exhibit increased pro-tumorigenic chemokines CCL20, CCL26, and CXCL8, alongside suppression of the ectopic receptor CCR10. This counterintuitive profile, where MS4A2 downregulation enhances TEM, suggests that mast cell dysregulation redirects lymphocyte trafficking toward the tumor parenchyma by disrupting peripheral homing signals^[29]^. Additionally, MS4A2 loss may trigger mast cell hyperactivation^[4,6]^, leading to heightened degranulation^[30,31]^ and release of proteases that degrade endothelial junction proteins and histamine that increases vascular permeability, potentially via IgE-independent pathways. These mechanisms explain the amplified TEM in MS4A2-low tumors, validated across independent cohorts.

The role of MS4A2 in mast cell regulation also links allergic inflammation to LUAD progression^[32]^. We previously reported that house dust mite, a common allergen, could induce lung cancer in mice^[18,33]^. However, the mechanistic connection between allergy and cancer remains unclear. MS4A2’s modulation of mast cell responses suggests it may mediate this relationship, warranting further exploration of allergen-driven oncogenesis.

The negative correlation between MS4A2 gene expression and sensitivity to 5-fluorouracil and axitinib suggests that clinicians should consider alternative treatments for LUAD patients with high MS4A2 expression, where these drugs may be less effective, and prioritize these agents for those with low expression, where sensitivity is higher. By using MS4A2 as a biomarker, treatment can be tailored to improve efficacy and patient outcomes, though its interplay with survival and immunity warrants further investigation for a holistic therapeutic strategy. The female-biased and stage-dependent regulation of MS4A2 highlights the potential for personalized immunotherapies, particularly for female patients with MS4A2-high tumors.

## Conclusions

Our bulk and single-cell RNA-Seq analyses highlight MS4A2 as a key immune regulator across cancers, with critical implications in LUAD. MS4A2 downregulation promotes mast cell-mediated leukocyte TEM in LUAD, reshaping immune infiltration and linking allergic inflammation to cancer progression. As a prognostic biomarker and therapeutic target, MS4A2 offers a roadmap for personalized interventions in a malignancy with limited treatment options^[32]^. By linking allergic inflammation to LUAD progression, MS4A2 connects immune responses to cancer, offering new ways to prevent tumor spread after surgery. Future research should validate these findings to bring MS4A2-based strategies into clinical practice, enhancing personalized care for LUAD patients. To propel these insights toward clinical translation, future studies could expand on current limitations as strategic opportunities. For example, validating MS4A2’s prognostic utility in multi-center cohorts with diverse LUAD subtypes and allergic comorbidities would strengthen its clinical relevance. Simultaneously, integrating spatial or time-resolved omics could map how MS4A2 dynamically orchestrates immune crosstalk across tumor niches. Importantly, emerging tools to resolve MS4A2 isoforms and post-translational modifications in mast cells may refine therapeutic targeting strategies. By embracing these challenges, the field can transform MS4A2’s exploratory potential into actionable precision medicine for LUAD interception.

## Data Availability

Datasets generated during and/or analyzed during the current study are publicly available.

## Research registration unique identifying number (UIN)

This study utilized publicly available data from The Cancer Genome Atlas (TCGA) database, and no new human participants were recruited. Therefore, registration in a public database was not applicable.
